# Quality Attributes of Ultra-High Temperature-Treated Model Beverages Prepared with Faba Bean Protein Concentrates

**DOI:** 10.3390/foods10061244

**Published:** 2021-05-30

**Authors:** Malik Adil Nawaz, Tanoj Kumar Singh, Regine Stockmann, Hema Jegasothy, Roman Buckow

**Affiliations:** 1 Commonwealth Scientific and Industrial Research Organisation (CSIRO), Agriculture and Food, 671 Sneydes Road, Private Bag 16, Werribee 3030, Australia; Tanoj.singh@csiro.au (T.K.S.); regine.stockmann@csiro.au (R.S.); hemalatha.jegasothy@csiro.au (H.J.); roman.buckow@sydney.edu.au (R.B.); 2 Centre for Advanced Food Engineering, School of Chemical and Biomolecular Engineering, The University of Sydney, Darlington 2008, Australia

**Keywords:** faba bean protein concentrate, legume-based beverage, colloidal stability, GC-MS

## Abstract

The objective of this research was to develop a model faba bean drink with a high concentration of protein (>4% *w/w*). The protein molecular weights and frequency for both faba and soy were assessed using SDS-PAGE. Results showed similarities in the protein molecular weight of both faba and soy (mainly 11S globulin ~Glycinin and 7S globulin ~β-conglycinin). Thus, faba can be considered as a potential soy replica in plant-based milk beverages. Oil-in-water emulsions (5–8% *w/w* available protein) were prepared using faba bean protein concentrate (FPC), 1% sunflower oil, and 0.2% sunflower lecithin. These emulsions were used as model beverages and were further investigated for UHT processibility, stability, and physicochemical properties. The physicochemical properties of emulsions at various processing stages viz., coarse emulsification, homogenisation, and UHT, were measured. An increase in the protein concentration and thermal treatment resulted in an increased oil droplet size, coalescence and flocculation, and protein aggregation. Lower protein concentrations viz., 5–6%, showed greater negative ζ-potential, and thereby, high dispersibility through enhanced electrostatic repulsions than those of higher concentrations (7–8%). Furthermore, an increase in protein concentration and UHT treatment resulted in an increased creaming index. In total, 21 different volatile compounds were detected and quantified, representing different chemical classes, namely alcohols, aldehydes, ketones, esters, furan, and acids. These volatiles have major consequences for the overall flavour chemistry of the model beverage product. Overall, this study showed the potential for application of faba bean as a protein source in UHT-treated legume-based beverages and identified areas for further development.

## 1. Introduction

Plant proteins have been an important component of human diets since ancient times and they appear in many traditional dishes and cuisines in the East-Asian and Indian populations (such as soy beverages, tofu, tempeh, and dosas) [1]. Industrialised food production has allowed for the mass production and enrichment/purification of plant proteins and new product innovation has transformed many of the traditional plant protein foods. For example, today, a wide range of commercial differentiated plant protein-based milks are available where previously only traditional soy, oat, rice, or coconut milks were regionally produced and consumed [2,3]. Further, plant proteins are increasingly used in the replacement of animal proteins in meat, dairy, and seafood products. In these food applications, plant proteins contribute to nutrition and provide physical functions such as solubility, emulsification, gelation, water, and fat-binding capacities [4]. These functionalities are critical to the successful application of plant proteins in redesigned animal products and are largely responsible for the stability, quality, and acceptability of the finished products [5].

A plant-based milk beverage is an oil-in-water (O/W) emulsion, where water is the aqueous phase and oil is the dispersed phase. The two phases are generally immiscible and are thermodynamically unstable, where they tend to separate overtime via creaming, flocculation, coalescence, and/or aggregation [3]. One of the critical functionalities of plant proteins is their ability to adsorb at the oil–water interface and form stabilising protein films around the oil droplets. Plant proteins that are readily adsorbed at the interface and are able to unfold their structures and form a cohesive film around the oil droplets should generally be considered as effective emulsifiers [6]. Many studies have evaluated the emulsifying properties of plant proteins such as soy [7], chickpea [8], peas [9], and beans [10,11], and demonstrated the potential of plant proteins to serve as stabilisers of O/W emulsions. Production of commercial plant protein-based beverages involves processing technologies such as high-shear mixing, homogenisation, and ultra-heat treatment (UHT). Such processing steps can create shear and thermal effects, which can lead to changes of the structure of proteins and protein assemblies, and subsequently affect the stability of the protein emulsions [12].

Among the plant proteins, faba bean (*Vicia faba*) protein is industrially underexploited as a food ingredient and primarily used as a fodder crop for animal consumption [13]. Faba bean, also known as fava bean or broad bean, is a legume rich in protein (250 g protein/kg seed). The major storage proteins consist of two globulin proteins, 11S (legumin) and 7S (vicilin) [14]. Both globulin proteins exhibit different physicochemical and functional properties [15]. Warsame et al. [16] showed that faba protein is comparable to other high-quality plant proteins such as soy; thus, it represents an emerging alternative source of plant protein. Though several basic studies have reported the emulsifying potential of faba beans in various emulsion systems, the efficacy of faba protein as an emulsifying agent in UHT-processed milk alternative beverages is still unexplored [11,17,18,19]. Felix et al. [17] evaluated the emulsifying properties of faba bean protein isolates (~89% purity) in an O/W emulsion system consisting of sunflower oil dispersed in the aqueous phase. The greatest stability and the smallest droplet sizes were obtained for the emulsion system created at pH 8.0 by high-shear mixing (18,000 rpm) and two-stages homogenisation (800 and 80 bar). Raikos et al. [18] reported that the emulsifying ability and stability of faba bean flour (composed of 300 g/kg protein) were superior when the emulsion was blended and homogenised (12,000 rpm) with flaxseed oil at pH 10. Liu et al. [19] studied the emulsifying properties of faba bean protein isolates by creating an emulsion system consisting of rapeseed oil dispersed in the aqueous dispersion of hydrolysed faba bean protein isolates. Karaca, Low, and Nickerson [10] prepared a series of emulsions by blending flaxseed oil with respective aqueous solutions of faba bean, chickpea, lentil, pea, and soy protein isolates, prepared with isoelectric precipitation and salt extraction. While all protein isolates prepared by isoelectric precipitation had superior emulsifying properties, the study also found similar emulsifying properties of faba bean protein isolates compared to soy and lentil protein isolates.

Plant-based beverages are becoming more popular and there is a large market of high-protein plant-based beverages. However, commercial products are inferior in nutritive value, especially in available protein and other functional ingredients such as dietary fibre and carbohydrates [20]. Industries usually prefer isolated protein as an ingredient to improve processibility and colloidal stability of the product [3]. This approach results in a stable but inferior nutritive quality product. Therefore, we explored the efficacy of faba bean protein concentrate as an ingredient in a faba bean drink protype. Additionally, most of the previous studies focused on emulsion stability using lower concentrations (<2%) of faba bean protein isolate. To our best knowledge, the efficacy of faba bean concentrate to prepare the O/W emulsions at a protein concentration above 3% and the colloidal stability of such emulsions after UHT preservation processing have not been investigated.

The objective of the current study was to develop a model faba bean drink with a high concentration of protein (>4% *w/w*) using faba bean protein concentrate. O/W emulsions (5–8% available protein) were prepared by blending sunflower oil, sunflower lecithin, and faba bean protein concentrates at a controlled pH condition followed by homogenisation and UHT processing. The emulsions created by high-shear mixing, homogenisation and ultra-heat thermal processing were evaluated for particle size, ζ-potential, (protein and lipid) droplet size, surface hydrophobicity, flocculation, coalescence, and creaming index. Headspace analysis of the high-protein emulsions at each processing step was performed to understand the formation of flavour compounds under different formulation and processing conditions.

## 2. Materials and Methods

### 2.1. Materials

Commercial faba bean protein concentrate (VITESSENCE^TM^ Pulse CT 3602 Protein) with 63% protein purity was purchased from Link Trading Queensland Pty Ltd., Richlands, QLD 4077, Australia. The faba protein concentrate on dry basis was carbohydrate (27% *w/w*), total fat (3% *w/w*), protein content (63% *w/w*), moisture content (3% *w/w*), total ash (4% *w/w*), and calories (410 KCal). Food-grade sunflower oil and soy lecithin were purchased from a local grocery store. Faba bean protein isolate (available protein ~ > 85%) and soy protein isolate (available protein ~ 83%) were produced in CSIRO pilot plant (Werribee, VIC, Australia). All other chemicals viz., potassium phosphate (food grade), sodium dodecyl sulphate (SDS) 8-anilino-1-naphthalenesulfonic acid (ANS), methanol, and Nile red dye were purchased from Sigma-Aldrich, Castle Hill NSW, Australia.

### 2.2. Sodium Dodecyl Sulphate Polyacrylamide Gel Electrophoresis (SDS-PAGE) of Faba and Soy Protein Isolates

Sodium dodecyl sulphate polyacrylamide gel electrophoresis (SDS-PAGE) is a non-continuous electrophoretic technique that is often used to isolate proteins with molecular masses ranging from 5 to 250 kDa. SDS-PAGE was performed on the Phast system (Pharmacia LKB Biotechnology) under reducing conditions. Mixtures of faba bean protein concentrate (FPC) having ~63% protein, faba bean protein isolate (FPI) having ~>85% protein, and soy protein isolate (SPI) having ~83% protein or reconstituted markers, were mixed with sample buffer and mercaptoethanol at a ratio 1:1:0.1, respectively, and heated in boiling water for 3 min followed by cooling in ice water. The treated samples/markers were loaded onto the gels (PhastGel gradient gels (8–25) purchased from GE Healthcare UK Ltd., Amersham Place, Little Chalfont, Buckinghamshire, HP7 9NA, United Kingdom) following the manufacturer’s protocol and electrophoresis was carried out at 60 Vh for 40 min using an automated program in the Phast gel system (PhastSystemTM, Amersham Biosciences UK Ltd., Amersham Place, Little Chalfont, Buckinghamshire, HP7 9NA, UK). Then the gels were immediately transferred to the development chamber for staining with PhastGel Blue stain followed by de-staining with acidic methanol and preserving with glycerol/acetic acid, using an automated method. The molecular weights (MWs) of unknown proteins were estimated via regression between the log of standard MWs and the relative mobility of the protein markers (GE Healthcare UK Ltd., Amersham Place, Little Chalfont, Buckinghamshire, HP7 9NA, UK).

### 2.3. Preparation of Emulsion

#### 2.3.1. Mixing

Faba bean concentrates at different protein concentrations (FPC5~5% *w/w*, FPC6~6% *w/w*, FPC7~7% *w/w*, and FPC8~8% *w/w*) were used in present study. Oil-in-water emulsions were prepared in 5 mM phosphate buffer, pH 8.0 (usually legume proteins have higher solubility at alkaline pH (8–9)). Therefore, the emulsions were prepared at pH 8, and in order to maintain the pH of the emulsion during processing, a phosphate buffer was used instead of deionized water. An appropriate amount of the protein concentrate, and sunflower lecithin (0.2% *w/w*) were mixed and hydrated with 5 mM phosphate buffer (pH 8.0) for 10 min at an operating temperature of ~40 °C using a high-shear lab mixer (Silverson L5M-A, Silverson Machines, Inc., East Longmeadow, MA, USA) at 1000 rpm. After hydration, the pH was readjusted to 8.0 using 0.1 N NaOH and the speed of the high-shear mixer was increased to 10,000 rpm and the sunflower oil (1% *w/w*) was added slowly (addition time ~20 min) to make a coarse emulsion (c-5, c-6, c-7, and c-8). The coarse emulsions were vacuum filtered using Whatman Grade 4 filter paper to remove unhydrated and/or particulated faba bean protein concentrate particles. No visible, unhydrated particles were observed on the filter for any of the coarse emulsion products. Therefore, no nitrogen measurement of the filter paper was performed.

#### 2.3.2. Preparation of Homogenised Emulsions

The coarse protein emulsions produced were further pressure homogenised (h-5, h-6, h-7, and h-8) to reduce the size of dispersed oil droplets. Each coarse emulsion was homogenised by single pass in an EmulsiFlex-C5 single-stage homogeniser (Avestin, Ottawa, Canada) operating at ~80 MPa and 25 °C.

#### 2.3.3. Ultra-High Temperature Processing of the Emulsion

The homogenised samples were subjected to UHT processing (uht-5, uht-6, uht-7, and uht-8) using a lab-scale UHT plant (FT74XA HTST/UHT System, Armfield, Hampshire, England). The thermal protocol consisted of preheating to 105 °C for 3 s and high-heat treatment at 135 °C for 3 s. The UHT plant was operated at a flow rate of 3 mL/s. When exposed to high temperature during UHT treatment, the homogenised emulsion samples did not foul the heating tubes during continuous processing over a 1 h time span. The flow rate, flow pressure, and outlet temperature were maintained at 3 ± 1 mL/s, 200 ± 20 kPa, and 135 ± 1 °C, respectively, during all runs.

**Figure 1:**
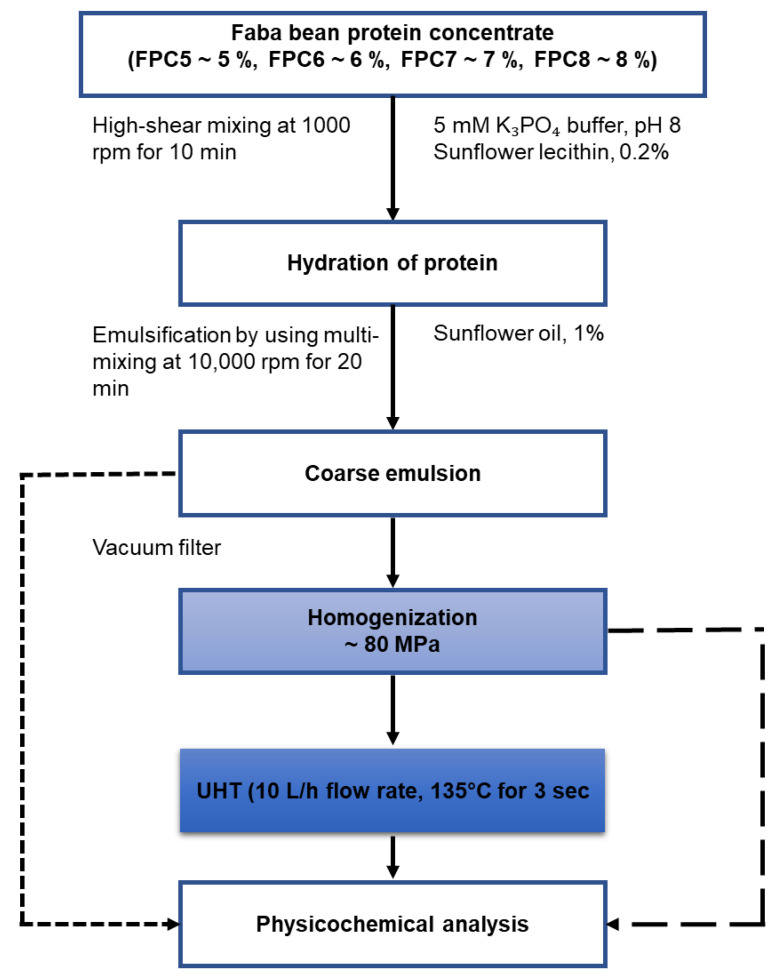
Schematic representation of the experimental process design for high concentration protein faba bean emulsions.

The schematic representation of experimental design of the current study is presented in Figure 1.

### 2.4. Physicochemical Properties of the Emulsion

#### 2.4.1. Particle Size Distribution

A Malvern Mastersizer was used to evaluate the average particle size distribution of the protein emulsions. All processed emulsions were immediately (~15 min) evaluated for particle size distribution. Milli-Q grade water (Refractive Index: 1.33) was used as the continuous phase of the experiment, whereas emulsion samples were used as the scattered phase. Particle size measurements were taken at approximately 12.5% laser obscuration, and the proportion of droplet size distributions at 10%, 50%, and 90% were d(0.1), d(0.5), and d(0.9), respectively. The uniformity, surface-weighted mean, D^3,2^, and volume-weighted mean, as well as D ^4,3^ of the particle size distribution graphs, were determined. [21]. The polydispersity index (PDI) was also calculated according to Equation (1).

PDI = d(0.9) − d(0.1)/d(0.5)(1)

#### 2.4.2. Flocculation Index (FI) and Coalescence Index (CI)

Flocculation is the mechanism by which undissolved particles bind together to create aggregates, thus destabilizing the emulsion. Coalescence is the mechanism by which two or more particles combine to create a single big particle as they come into contact [22]. All processed emulsion samples were immediately (~15 min) evaluated for flocculation and coalescence indexes (FI and CI, respectively). The FI and CI of the emulsion samples were calculated according to the method of Felix et al. [23]. Emulsions were diluted (1:10) in deionised water (DI) or 1% sodium dodecyl sulphate (SDS), and D ^4,3^ values of freshly prepared and stored (after 24 h) emulsions were determined using the Mastersizer. To determine the CI, both emulsions were dissolved in a 1% SDS solution.

The FI and CI were calculated by Equations (2) and (3), respectively.

(2) $$\mathrm{FI}\;\left(\%\right)=\left[D^{4,3}\;\mathrm{in}\;\mathrm{water}-1\right]\times \frac{100}{D^{4,3}\;\mathrm{in}\;\mathrm{SDS}}$$

(3) $$\mathrm{CI}\;\left(\%\right)=\left[D^{4,3}\;\left(24\;h\right)-1\right]\times \frac{100}{D^{4,3}\;\left(0\;h\right)}$$

where D ^4,3^ in water is sample diluted in water, D ^4,3^ in SDS is the sample diluted in sodium dodecyl sulphate (SDS), D ^4,3^ (24 h) is the sample stored for 24 h in the refrigerator (~4 °C), and D ^4,3^ (0 h) is the freshly prepared samples.

#### 2.4.3. Confocal Laser Scanning Microscopy

The emulsions were analysed microscopically by labelling fat drops with Nile Red and then using confocal laser scanning microscopy (CLSM), as reported by Qamar, Bhandari, and Prakash [12]. Briefly, a 0.5 mL aliquot of protein emulsion and 2 mL of Nile Red (0.02% *w/v* in polythene glycol) were correctly mixed and held for 5 min in the dark. A drop of the mixture was mounted on a glass slide for the purpose of determining the size of the lipid droplets in the study.

#### 2.4.4. ζ-Potential

The ζ-potential of the samples was determined by Malvern Zetasizer as described by Li et al. [24]. Briefly, freshly processed coarse, homogenised, and UHT-treated samples were prediluted 10-fold in a 5 mM solution of phosphate buffer (pH 8.0) prepared with Milli-Q grade water (to minimise the artifact of other minor constituents of the model beverage [25]) and transferred to a ζ-potential cell. At a refractive index of 1.33, the ζ-potential was estimated.

#### 2.4.5. Creaming Index

The creaming index of the freshly processed emulsions was calculated by centrifuging 5 mL of the emulsion in a plastic centrifuge tube (at 20 °C and 4000 rpm for 20 min) using the modified method of Liu et al. [26]. Each tube was then refrigerated at ~ 4° C for 1 week. After 1 week of undisturbed storage, the total height of emulsion (H_t_) and height of cream (H_c_) were estimated. The creaming index was calculated by using Equation (4).

(4) $$\mathrm{Creaming}\;\mathrm{index}=\left(\frac{H_{t}}{H_{c}}\right)\times 100$$

#### 2.4.6. Headspace Gas Chromatography-Mass Spectrometry (GC-MS)

Aliquots (~5 g) of selected samples of protein emulsions prepared fresh and UHT samples stored for one month at refrigerated temperature (~4 °C) were weighed in amber 22 mL headspace vials and analysed for volatile organic compounds (VOCs). The analysis was performed using an Agilent gas chromatography mass spectrometry system (GCMS; 6890N model GC and 5975B model MSD; Agilent Technologies Australia Pty Ltd., Mulgrave, Victoria, Australia) equipped with a CombiPAL robotic autosampler (CTC Analytics AG, Zwingen, Switzerland). Volatiles in the sample vial were extracted/concentrated from headspace on SPME fibre (carboxen/polydimethylsiloxane, CAR/PDMS; 85 μm film thickness; 10 mm long; Supelco, Bellefonte, PA) for 30 min at 50 °C. SPME-sampled volatiles were thermally desorbed directly in the GC inlet (split less mode, 250 °C, 1 min) and chromatographed on a VF-WAXms column (30 m × 0.32 mm × 1.0 μm; Agilent Technologies) using a temperature gradient. Temperature increased from 35 to 225 °C at 10 °C/min with initial and final hold time of 3 and 10 min, respectively. Eluted compounds were detected by the GCMS system interfaced with the computer using MassHunter Workstation software (version 10.0; Agilent Technologies). Details on the set-up of GCMS, identification, and quantification of compounds were followed as detailed elsewhere [27,28].

### 2.5. Statistical Analysis

All experiments were performed in triplicate, and the results were expressed in means ± standard deviations. Experimental data were assessed by one-way analysis of variance (ANOVA) with Minitab 19 (Minitab^®^ for Windows Release 19, Minitab Inc, Chicago, IL, USA) to determine the significant differences. The data were then analysed using Tukey’s pair-wise comparison, at a 5% level of significance, to compare the results between different treatments. Principal component analysis (PCA) of quantitative data from volatile analysis was performed using XLStat by Addinsoft (add on to Microsoft Excel).

## 3. Results and Discussion

### 3.1. Sodium Dodecyl Sulphate Polyacrylamide Gel Electrophoresis (SDS-PAGE) of Faba and Soy Protein Isolates

**Figure 2:**
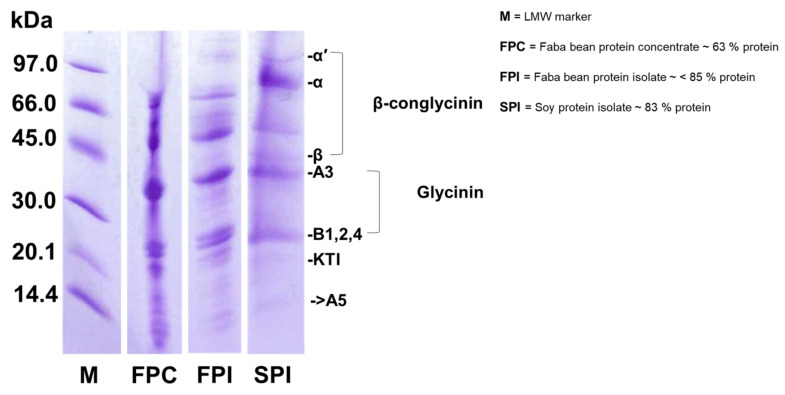
SDS-PAGE patterns of low molecular weight marker (M), faba bean protein concentrate (FPC), faba bean protein isolate (FPI), and soy protein isolate (SPI).

The SDS-PAGE for faba bean protein concentrate (FPC) having 63% protein, faba bean protein isolate (FPI) having >85% protein, and soy protein isolate (SPI) having 83% protein is presented in Figure 2. Various distinct protein bands were obtained from faba and soy protein isolates with MW ranging from ~14 to 97 kDa. The molecular weights for 7S proteins are between 45 and 97 kDa, and for 11S they are below 36 kDa [29]. These included major seed storage proteins such as legumin, vicilin, and convicilin, as well as other protein classes like lipoxygenase, heat shock proteins, sucrose-binding proteins, albumin, and defensin [30,31]. Results showed similarities in the protein molecular weights and frequency of both faba and soy; therefore, we hypothesized that faba protein could be used as an alternative to soy in plant-based protein milk emulsions, and this research aimed to investigate the processibility and functionality of emulsions made from faba bean concentrate.

### 3.2. Physicochemical Properties of the Emulsions

#### 3.2.1. Particle Size Distribution

**Figure 3:**
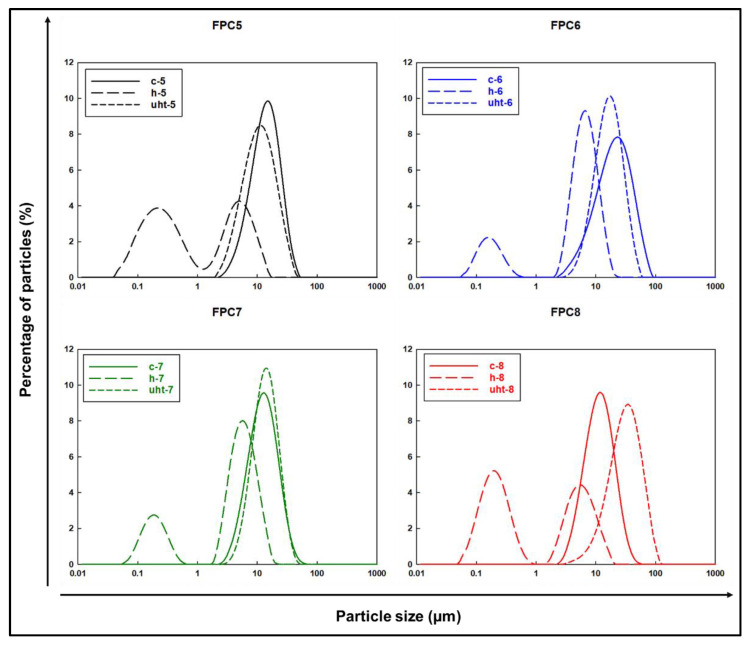
Particle size distribution curves of O/W emulsions with various concentrations of faba bean protein concentrate at various stages during UHT processing.

The particle size distribution of coarse (c-5, c-6, c-7, and c-8), homogenised (h-5, h-6, h-7, and h-8), and UHT (uht-5, uht-6, uht-7, and uht-8)-treated emulsions of faba bean protein concentrate are presented in Figure 3 and Table 1. The D ^4,3^ and D ^3,2^ are volume or mass moment means (De Brouckere Mean Diameter) and surface area moment means (Sauter Mean Diameter), respectively. The uniformity and polydispersity index was also measured for the geometrical size distribution of particles in the emulsion. Results showed that the UHT treatment resulted in increased size of particles probably due to thermally induced aggregation of proteins on the surface of droplets, which may be overcome by introducing low-pressure homogenisation after the heat treatment. The limitation of the particle size distribution analysis is to distinguish between disperse particles, as the detection range of the equipment used is between 0.1 and 1000 µm. In this range, it is likely that the particle size analysis includes not only fat globules, but also some protein aggregates and even protein-fat globules and globule aggregates [32].

Homogenisation of emulsions results in cavitation, which disrupts electrostatic and hydrophobic interactions in proteins, avoiding aggregation and facilitating the formation of small particles [33], which is evident by the lower D ^4,3^ values of the homogenised emulsion compared to the coarse and UHT-treated emulsions (Table 1). Contrarily, the increase in protein concentration from 5% to 8% resulted in more unabsorbed protein in the serum phase, which denatured during UHT and possibly interacted with the interface, resulting in a significant (*p* > 0.05) increase in the D ^4,3^ values of UHT-treated emulsions. In addition, the serum phase is expected to contain significant levels of polysaccharides (primarily starch), which could also gelatinise during thermal applications and resulted in increased particle size and consequently increased viscosity [34].

The particle size distribution graphs of the processed emulsions are presented in Figure 3. The distribution of coarse and UHT emulsions ranged between 1 and 100 µm, while for homogenised emulsions it was between 0.01 and 100 µm. To measure the different sizes of the particles in processed emulsions and the light scattering intensity of these particles, the uniformity and polydispersity index (PDI) was also calculated (Table 1). It can be noted in Table 1 that the coarse and UHT emulsion with increasing protein from 5% to 8% had a relatively increasing trend in uniformity and PDI. This suggests that the emulsion with higher protein had larger particles and lower dynamic light scattering properties compared lower protein counterparts.

#### 3.2.2. Flocculation Index (FI) and Coalescence Index (CI)

**Figure 4:**
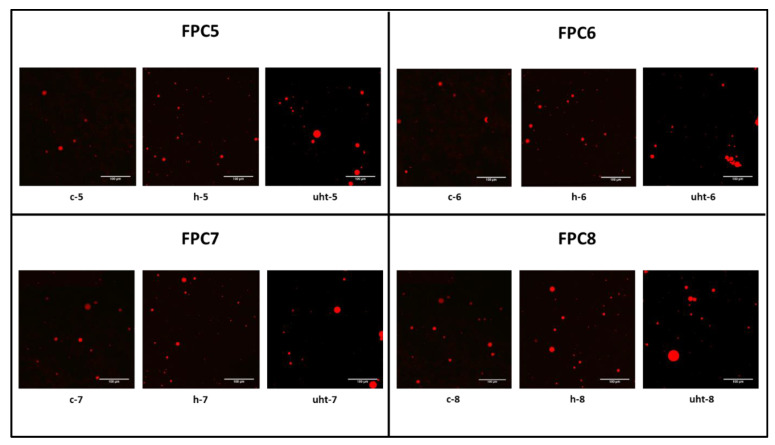
Confocal laser scanning micrographs of O/W emulsions with various concentrations of faba bean protein at various stages during UHT processing. Scale bar on each image = 100 µm.

Emulsions are very complex systems with oil as a dispersed phase in an aqueous continuous phase [35]. They are thermodynamically unstable, but depending on their composition and processing, they can become kinetically stable [36]. Several critical variables in emulsion preparation include the solubility of the two phases, the quantity and type of surfactants and/or protein used, and the volume ratio of the continuous and dispersed phases [36,37]. In emulsions, a variety of destabilization processes may occur, including one or more of the following phenomena: droplet movement, accumulation, and droplet size increase. This irreversible enlargement of droplet may occur through distinct mechanisms viz., Ostwald ripening, flocculation, and coalescence. Ostwald ripening usually takes place in water-in-oil emulsions and involves a diffusive transfer of the dispersed phase from smaller to larger droplets. Conversely, flocculation usually takes place in oil-in-water emulsions. It is a process by which undissolved particles accumulate and destabilize the emulsion [38,39]. Coalescence is the process by which two or more particles collide to form a single large particle [40]. Thus, in the current study, FI and CI are used as the indicative assessment of emulsion stability. The results of FI and CI for coarse (c-5, c-6, c-7, and c-8), homogenised (h-5, h-6, h-7, and h-8), and UHT (uht-5, uht-6, uht-7, and uht-8)-treated emulsions of faba bean protein concentrate are presented in Table 1. Results showed that the FI and CI of emulsion varied with not only the protein concentration, but also the processing stage. An increase in the protein concentration and thermal treatment during UHT processing resulted in an increased droplet flocculation [41]. The interactions between adsorbed proteins inside a droplet or between droplets can influence the emulsions’ viscosity. The bridge formation between droplets, as well as the existence of droplets with huge aggregates on their surfaces, also contributes to an increase in emulsion viscosity [42]. Rheological experiments were not carried out on our emulsions as particle size analysis already indicated the presence of agglomerated oil droplets. Similar findings were observed in confocal laser scanning micrographs (CLSM) of coarse (c-5, c-6, c-7, and c-8), homogenised (h-5, h-6, h-7, and h-8), and UHT (uht-5, uht-6, uht-7, and uht-8)-treated emulsions of faba bean protein concentrate, which are presented in Figure 4.

#### 3.2.3. ζ-Potential

ζ-potential is useful in predicting emulsion stability. It is now generally understood that the emulsions having low electronegativity are unstable and easily flocculate or coagulate during storage, while the emulsions with more electronegativity are comparatively more stable due to more repulsive forces than attractive forces [43]. The ζ-potential of coarse (c-5, c-6, c-7, and c-8), homogenised (h-5, h-6, h-7, and h-8), and UHT (uht-5, uht-6, uht-7, and uht-8)-treated emulsions of faba bean protein concentrate are presented in Table 1. Results showed that the ζ-potential not only depended on the concentration of protein in emulsion, but also on the processing stage. Similar findings are reported by Delahaije et al. [44], who found that the electronegativity of a protein is a function of its amino acid sequence and pH/ionic strength of the matrix. Low-protein concentrations viz., FPC5 and FPC6, showed a greater negative ζ-potential, and thereby high solubility, through enhanced electrostatic repulsions than that of FPC7 and FPC8. Additionally, the processing stage, especially UHT treatment, resulted in less negative values compared to coarse and homogenised emulsions. This can be related to the formation of protein aggregates that mainly occurs at a higher temperature, which resulted in a reduction of protein solubility. Similar findings were recorded in particle size analysis (Figure 3) and CLSM (Figure 4), where increased mean particle size was detected.

#### 3.2.4. Creaming Index

Creaming index or gravitational separation is also an indication of emulsion stability [45]. The creaming indexes of coarse (c-5, c-6, c-7, and c-8), homogenised (h-5, h-6, h-7, and h-8), and UHT (uht-5, uht-6, uht-7, and uht-8)-treated emulsions of faba bean protein concentrate are presented in Table 1. Results showed that the creaming index not only depended on the concentration of protein in emulsions, but also on the processing stage. Higher protein concentrations viz., FPC7 and FPC8, showed higher creaming index compared to lower protein emulsions viz., FPC5 and FPC6. Previous studies also reported the increased creaming index of the emulsions with an increase of plant proteins [12,45].

The creaming index of the homogenised emulsions was significantly (*p* > 0.05) lower than that of coarse emulsions. Tangsuphoom and Coupland [46] reported a similar trend of decreased creaming index of homogenised coconut milk compared to the unhomogenised counterpart. They further concluded that the reduced particle size of homogenized coconut milk might resist the separation of cream. Additionally, thermal application during UHT processing induced increased cream separation in all treatments.

#### 3.2.5. Headspace Gas Chromatography-Mass Spectrometry (GC-MS)

Volatile compounds profiling of products at each of the above three steps of manufacture of model faba protein-based beverage was performed by the sampling of volatile compounds using SPME followed by GCMS analysis. In total, 21 compounds were detected and quantified, representing different chemical classes, namely alcohols, aldehydes, ketones, esters, furan, and acids. These volatiles have major consequences for the overall flavour chemistry of the model beverage product.

**Figure 5:**
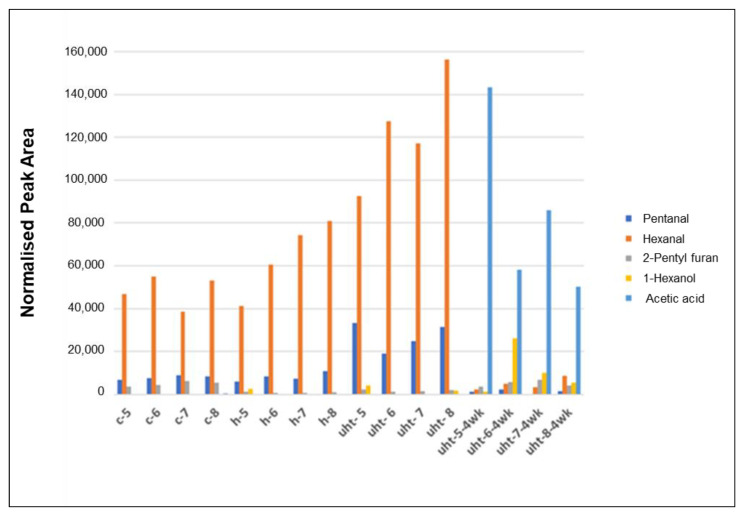
Changes in the concentration of key volatile organic compounds during manufacture of the model faba protein-based beverage and subsequent storage. Three distinct steps were as follows: (1) preparation of coarse emulsion (c-5 to c-8), (2) homogenization of coarse emulsion (h-5 to h-8), and (3) thermal stabilization of the model beverage by UHT treatment (uht-5 to uht-8). Thermally stabilized beverages were stored at 4 °C for 4 weeks.

Origins of a majority of these volatile organic compounds can be attributed to oxidative deterioration of lipids. Several lipid oxidation-derived compounds were known potent odorants with extremely low human sensory detection thresholds, namely pentanal, hexanal, heptanal, nonanal, *E*-2-heptenal, and 2-pentyl furan. Odour notes mainly attributed to these compounds are green, grassy, leafy, cardboard, beany, rancid, etc. These compounds are primarily oil soluble and known to be adsorbed on to the proteins. The concentration of volatiles in general increased at each of the steps involved in the manufacture of the model beverage (Figure 5). Lipids in the model faba protein-based beverage were primarily composed of sunflower oil (1%), sunflower lecithin (0.2%), and residual lipids in the faba protein preparation. Both faba bean and sunflower oil contained high levels of unsaturated fatty acids, where up to 95% of total fatty acid profiles were represented by oleic (C18:1, ~15%) and linoleic (C18:2, ~70%) acids [47,48]. Sunflower oil and lecithin used in the preparation of the beverage showed no sign of oxidative deterioration with the absence of above-mentioned volatile compounds. In the model plant protein beverage described above, the volatile compounds found their way in from the faba protein concentrate, where these compounds were most likely produced via enzymatic and/or auto-oxidation of faba bean lipids occurring during the processing, production, and storage/distribution of the plant protein ingredient from faba beans. This is a common problem associated with most commercial protein concentrates and isolates. Lipid oxidation-derived volatile compounds present in faba protein concentrate, used in the present study, imparted a distinct oxidised/bean odour note. The increase in the levels of volatiles, during the manufacture of model beverage, could be attributed to the thermal degradation of lipid oxidation-derived intermediate compounds, namely hydroperoxides of various unsaturated fatty acids. Nonanal most likely originated from the oxidation of oleic acid present in model beverage lipids. However, the origins of the other volatile compounds, namely pentanal, hexanal, heptanal, *E*-2-heptenal, and 2-pentyl furan, could mainly be attributed to the oxidation of linoleic acid (C18:2), which was the most dominant fatty acid in faba and sunflower oils. Sensory detection thresholds for the lipid oxidation-derived volatiles identified in the present study, namely pentanal, hexanal, heptanal, nonanal, *E*-2-heptenal, and 2-pentyl furan, were in extremely low parts per million (ppm or mg kg^-1^) as reported in numerous studies.

**Figure 6:**
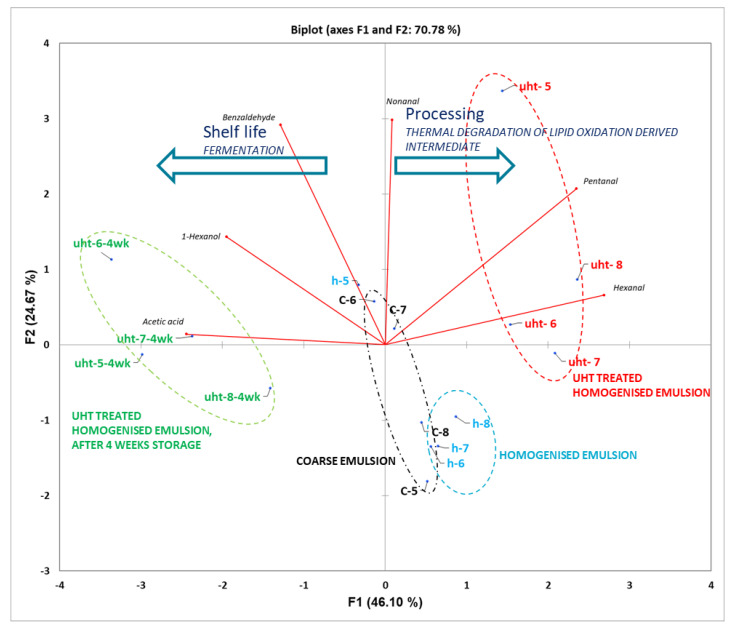
Principal component analysis of quantitative data from volatile analysis on model beverages during the manufacture of a model faba protein-based beverage and subsequent storage; see Figure 5 for sample details. Key groupings of samples were highlighted as (i) various processing steps involved and (ii) underlying biochemical changes occurring during the preparation and storage of the UHT-treated model faba protein-based beverage.

Volatile profiling of thermally stabilised samples of the model beverages stored for 4 weeks at a refrigerated temperature clearly showed the formation of microbial/fermentation activity with the production of elevated levels of acetic acid. In addition, it was also noted that levels of key lipid oxidation-derived volatile aldehydes, e.g., pentanal, hexanal, etc., significantly reduced with the concomitant increase in the levels of corresponding alcohols like 1-pentanol and 1-hexanol. This reduction in aldehyde could be attributed to reductive enzymes of microbial cells. Principal component analysis was performed on the volatile quantitative data in order to elucidate underlying trends/pattern (Figure 6). PCA biplot captured 70% of the underlying variation in the data and clearly highlighted key biochemical reaction mechanisms involved in production and storage, namely lipid oxidation and fermentation/microbial degradation during production/processing and storage, respectively. The right half of the PCA biplot clearly showed distinct grouping for the coarse emulsion, and as it was further processed by homogenization and UHT treatment, it resulted in an increase in the levels of aldehydes, produced from thermal degradation of lipid oxidation-derived hydroperoxide. The samples of model faba protein-based beverage stored at a refrigerated temperature for one month were grouped together in the left half of the PCA biplot clearly highlighting that the underlying changes were negatively correlated with the disappearance or conversion of major lipid oxidation-derived aldehydes, namely pentanal and hexanal, into corresponding alcohols by the enzymatic reduction by microbes. In addition, microbial fermentation of sugars led to the production of high levels of acetic acid.

## 4. Conclusions

The faba bean is one of the emerging sources of plant proteins. In the present study, SDS-PAGE of faba and soy protein concentrates and isolates showed that the protein molecular weights of faba are similar to their soy counterparts. Thus, faba proteins are promising alternatives to soy proteins in plant-based beverages. We found that emulsions prepared with 5% to 8% faba bean protein concentrate and then subjected to UHT treatment were unstable, as indicated by increased droplet aggregation, the creaming index, and flocculation. UHT processing clearly affected the volatile flavour profile of faba bean model beverages with 4-week storage inducing further changes. Reformulation of protein and starch content in the faba bean protein, as well as optimization of the emulsification and UHT procedures, is required to achieve stable faba bean beverage systems; this study showed the potential for the application of faba beans as a protein source in UHT-treated legume-based beverages and identified areas for further development.

## Figures and Tables

**Table 1:** Effect of faba bean protein concentration on the physicochemical properties viz., particle size distribution, polydispersity index (PDI), flocculation index (FI), coalescence index (CI), ζ-potential, and creaming index of O/W emulsions at various stages during UHT processing *.

Treatment	Emulsion Type	Particle Size Distribution				PDI (%)	FI	CI	ζ-Potential (mV)	Creaming Index
		D ^4,3^–Volume-Weighed Mean (µm)	Uniformity (µm)	Specific Surface Area (µm)	D ^3,2^–Surface-Weighed Mean (µm)					
FPC5	coarse	14.39 ± 0.00 ^c,d^	0.45 ± 0.00 ^b,c,d^	0.56 ± 0.01 ^e,f,g^	10.71 ± 0.00 ^c^	24.38 ± 0.01 ^c^	88.81 ± 0.35 ^d^	101.26 ± 5.24 ^c,d^	−27.60 ± 3.25 ^d^	12.45 ± 0.35 ^b^
	homogenised	2.48 ± 0.04 ^f^	0.23 ± 0.02 ^d^	21.50 ± 0.14 ^a^	0.28 ± 0.00 ^f^	6.88 ± 0.24 ^e^	4.79 ± 0.19 ^e^	63.93 ± 0.34 ^e,f^	−24.63 ± 1.50 ^c,d^	7.00 ± 0.28 ^g^
	UHT	11.83 ± 0.16 ^c,d^	0.45 ± 0.02 ^b,c,d^	0.65 ± 0.01 ^e,f^	9.24 ± 0.20 ^d^	21.37 ± 0.71 ^c,d^	105.06 ± 3.66 ^d^	142.34 ± 9.92 ^b^	−23.10 ± 0.96 ^b,c^	9.25 ± 021 ^d,e^
FPC6	coarse	22.19 ± 2.85 ^b^	0.57 ± 0.01 ^a,b^	0.44 ± 0.02 ^g,h^	13.57 ± 0.60 ^b^	41.52 ± 5.45 ^b^	160.82 ± 17.80 ^c^	101.57 ± 2.24 ^c,d^	−23.10 ± 2.10 ^b,c^	13.05 ± 0.21 ^b^
	homogenised	5.55 ± 1.52 ^e,f^	0.70 ± 0.20 ^a^	9.17 ± 0.05 ^b^	0.50 ± 0.22 ^f^	10.44 ± 0.13 ^d,e^	11.10 ± 2.87 ^e^	47.81 ± 0.49 ^f^	−21.20 ± 0.80 ^b^	7.20 ± 0.42 ^g^
	UHT	17.25 ± 0.92 ^b,c^	0.44 ± 0.00 ^b,c,d^	0.45 ± 0.01 ^g,h^	13.38 ± 0.29 ^b^	29.03 ± 1.81 ^c^	194.98 ± 0.15 ^a,b^	175.32 ± 4.81 ^a^	−21.47 ± 1.10 ^b,c^	10.25 ± 0.50 ^c,d^
FPC7	coarse	10.69 ± 0.26 ^d,e^	0.44 ± 0.00 ^b,c,d^	0.73 ± 0.01 ^e^	8.26 ± 0.08 ^d^	22.81 ± 0.33 ^c^	182.22 ± 12.42 ^b,c^	110.95 ± 5.30 ^c^	−0.02 ± 0.02 ^a^	14.55 ± 0.21 ^a^
	homogenised	4.65 ± 1.79 ^f^	0.37 ± 0.01 ^b,c,d^	1.16 ± 0.04 ^d^	5.18 ± 0.15 ^e^	9.17 ± 0.41 ^e^	10.83 ± 3.06 ^e^	83.41 ± 7.59 ^d,e^	0.08 ± 0.03 ^a^	7.95 ± 0.21 ^f,g^
	UHT	14.08 ± 0.29 ^c,d^	0.39 ± 0.02 ^b,c,d^	0.53 ± 0.01 ^f,g^	11.26 ± 0.14 ^c^	22.89 ± 1.45 ^c^	212.14 ± 3.91 ^a,b^	191.81 ± 11.82 ^a^	−0.12 ± 0.07 ^a^	10.40 ± 0.28 ^c^
FPC8	coarse	12.22 ± 0.10 ^c,d^	0.48 ± 0.00 ^a,b,c^	0.66 ± 0.00 ^e,f^	9.11 ± 0.07 ^d^	20.89 ± 0.18 ^c,d^	199.74 ± 18.27 ^a,b^	109.06 ± 1.30 ^c^	0.01 ± 0.07 ^a^	14.95 ± 0.21 ^a^
	homogenised	2.87 ± 0.01 ^f^	0.28 ± 0.00 ^c,d^	4.92 ± 0.01 ^c^	1.22 ± 0.00 ^f^	7.84 ± 0.34 ^e^	16.10 ± 1.27 ^e^	78.98 ± 0.32 ^d,e^	0.01 ± 0.20 ^a^	8.40 ± 0.14 ^e,f^
	UHT	33.27 ± 3.55 ^a^	0.49 ± 0.02 ^a,b,c^	0.27 ± 0.01 ^h^	22.11 ± 0.78 ^a^	59.49 ± 7.40 ^a^	226.50 ± 0.26 ^a^	197.43 ± 7.08 ^a^	0.24 ± 0.11 ^a^	15.15 ± 0.21 ^a^

⁎ Values (mean ± S.D.) are in triplicates (*n* = 3) and the values within a column followed by the same letter are not significantly different at 95% confidence level (*p* < 0.05)

## References

[1] Young V.R., Pellett P.L. (1994). Plant proteins in relation to human protein and amino acid nutrition. Am. J. Clin. Nutr..

[2] McClements D.J., Newman E., McClements I.F. (2019). Plant-based Milks: A Review of the Science Underpinning Their Design, Fabrication, and Performance. Compr. Rev. Food Sci. Food Saf..

[3] Nawaz M.A., Tan M., Øiseth S., Buckow R. (2020). An Emerging Segment of Functional Legume-Based Beverages: A Review. Food Rev. Int..

[4] Li-Chan E.C.Y., Lacroix I.M.E., Yada R.Y. (2018). 1—Properties of proteins in food systems: An introduction. Proteins in Food Processing.

[5] Queirós R.P., Saraiva J.A., da Silva J.A.L. (2018). Tailoring structure and technological properties of plant proteins using high hydrostatic pressure. Crit. Rev. Food Sci. Nutr..

[6] Damodaran S. (2005). Protein Stabilization of Emulsions and Foams. J. Food Sci..

[7] Fernandez-Avila C., Trujillo A.J. (2016). Ultra-High Pressure Homogenization improves oxidative stability and interfacial properties of soy protein isolate-stabilized emulsions. Food Chem..

[8] Wang S., Chelikani V., Serventi L. (2018). Evaluation of chickpea as alternative to soy in plant-based beverages, fresh and fermented. LWT.

[9] Chao D., Aluko R.E. (2018). Modification of the structural, emulsifying, and foaming properties of an isolated pea protein by thermal pretreatment. CyTA J. Food.

[10] Karaca A.C., Low N., Nickerson M. (2011). Emulsifying properties of chickpea, faba bean, lentil and pea proteins produced by isoelectric precipitation and salt extraction. Food Res. Int..

[11] Tsoukala A., Papalamprou E., Makri E., Doxastakis G., Braudo E.E. (2006). Adsorption at the air–water interface and emulsification properties of grain legume protein derivatives from pea and broad bean. Colloids Surf. B Biointerfaces.

[12] Qamar S., Bhandari B., Prakash S. (2019). Effect of different homogenisation methods and UHT processing on the stability of pea protein emulsion. Food Res. Int..

[13] Crépon K., Marget P., Peyronnet C., Carrouée B., Arese P., Duc G. (2010). Nutritional value of faba bean (*Vicia faba* L.) seeds for feed and food. Field Crop. Res..

[14] Multari S., Stewart D., Russell W.R. (2015). Potential of Fava Bean as Future Protein Supply to Partially Replace Meat Intake in the Human Diet. Compr. Rev. Food Sci. Food Saf..

[15] Kimura A., Fukuda T., Zhang M., Motoyama S., Maruyama N., Utsumi S. (2008). Comparison of Physicochemical Properties of 7S and 11S Globulins from Pea, Fava Bean, Cowpea, and French Bean with Those of Soybean—French Bean 7S Globulin Exhibits Excellent Properties. J. Agric. Food Chem..

[16] Warsame A.O., Michael N., O’Sullivan D.M., Tosi P. (2020). Identification and quantification of major faba bean seed proteins. J. Agric. Food Chem..

[17] Felix M., Cermeño M., FitzGerald R.J. (2019). Assessment of the microstructural characteristics and the in vitro bioactive properties of sunflower oil-based emulsions stabilized by fava bean (vicia faba) protein. Food Hydrocoll..

[18] Raikos V., Neacsu M., Russell W., Duthie G. (2014). Comparative study of the functional properties of lupin, green pea, fava bean, hemp, and buckwheat flours as affected by pH. Food Sci. Nutr..

[19] Liu C., Bhattarai M., Mikkonen K.S., Heinonen M. (2019). Effects of Enzymatic Hydrolysis of Fava Bean Protein Isolate by Alcalase on the Physical and Oxidative Stability of Oil-in-Water Emulsions. J. Agric. Food Chem..

[20] Vogelsang-O’Dwyer M., Sahin A.W., Zannini E., Arendt E.A. (2021). Physicochemical and nutritional properties of high protein emulsion-type lupin-based model milk alternatives: Effect of protein source and homogenisation pressure. J. Sci. Food Agric..

[21] Rahmati N.F., Koocheki A., Varidi M., Kadkhodaee R. (2018). Thermodynamic compatibility and interactions between Speckled Sugar bean protein and xanthan gum for production of multilayer O/W emulsion. J. Food Sci. Technol..

[22] Oliete B., Potin F., Cases E., Saurel R. (2019). Microfluidization as Homogenization Technique in Pea Globulin-Based Emulsions. Food Bioprocess. Technol..

[23] Felix M., Cermeño M., Romero A., FitzGerald R.J. (2019). Characterisation of the bioactive properties and microstructure of chickpea protein-based oil in water emulsions. Food Res. Int..

[24] Li Q., Wang Z., Dai C., Wang Y., Chen W., Ju X., Yuan J., He R. (2019). Physical stability and microstructure of rapeseed protein isolate/gum Arabic stabilized emulsions at alkaline pH. Food Hydrocoll..

[25] Lu G.W., Gao P., Kulkarni V.S. (2010). CHAPTER 3—Emulsions and Microemulsions for Topical and Transdermal Drug Delivery. Handbook of Non-Invasive Drug Delivery Systems.

[26] Liu N., Chen Q., Li G., Zhu Z., Yi J., Li C., Chen X., Wang Y. (2018). Properties and Stability of Perilla Seed Protein-Stabilized Oil-in-Water Emulsions: Influence of Protein Concentration, pH, NaCl Concentration and Thermal Treatment. Molecules.

[27] Gao L., Chen W., Xu X., Zhang J., Singh T.K., Liu S., Zhang D., Tian L., White A., Shrestha P. (2020). Engineering trienoic fatty acids into cottonseed oil improves low-temperature seed germination, plant photosynthesis and cotton fibre quality. Plant. Cell Physiol..

[28] Torkamani A.E., Juliano P., Ajlouni S., Singh T.K. (2014). Impact of ultrasound treatment on lipid oxidation of Cheddar cheese whey. Ultrason. Sonochemistry.

[29] Wu W., Zhang C., Kong X., Hua Y. (2009). Oxidative modification of soy protein by peroxyl radicals. Food Chem..

[30] Hosseini A., Jafari S.M., Mirzaei H., Asghari A., Akhavan S. (2015). Application of image processing to assess emulsion stability and emulsification properties of Arabic gum. Carbohydr. Polym..

[31] Liu S., Zhou R., Tian S., Gai J. (2007). A study on subunit groups of soybean protein extracts under SDS-PAGE. J. Am. Oil Chem. Soc..

[32] Ahmadian-Kouchaksaraei Z., Varidi M., Varidi M.J., Pourazarang H. (2014). Influence of processing conditions on the physicochemical and sensory properties of sesame milk: A novel nutritional beverage. LWT Food Sci. Technol..

[33] O’Sullivan J., Murray B., Flynn C., Norton I. (2016). The effect of ultrasound treatment on the structural, physical and emulsifying properties of animal and vegetable proteins. Food Hydrocoll..

[34] Boode K., Walstra P., de Groot-Mostert A.E.A. (1993). Partial coalescence in oil-in-water emulsions 2. Influence of the properties of the fat. Colloids Surf. A Physicochem. Eng. Asp..

[35] McClements D.J. (2015). Food Emulsions: Principles, Practices, and Techniques.

[36] Bibette J., Calderon F.L., Poulin P. (1999). Emulsions: Basic principles. Rep. Prog. Phys..

[37] El-Jaby U., Cunningham M., McKenna T.F.L. (2009). Comparison of emulsification devices for the production of miniemulsions. Ind. Eng. Chem. Res..

[38] Hubbard A.T. (2002). Encyclopedia of Surface and Colloid Science.

[39] Santos J., Calero N., Trujillo-Cayado L.A., Garcia M.C., Muñoz J. (2017). Assessing differences between Ostwald ripening and coalescence by rheology, laser diffraction and multiple light scattering. Colloids Surf. B Biointerfaces.

[40] Ralla T., Salminen H., Braun K., Edelmann M., Dawid C., Hofmann T., Weiss J. (2020). Investigations into the Structure-Function Relationship of the Naturally-Derived Surfactant Glycyrrhizin: Emulsion Stability. Food Biophys..

[41] Dickinson E. (2010). Flocculation of protein-stabilized oil-in-water emulsions. Colloids Surf. B Biointerfaces.

[42] Keerati-u-rai M., Corredig M. (2009). Heat-induced changes in oil-in-water emulsions stabilized with soy protein isolate. Food Hydrocoll..

[43] Pinto I., Buss A. (2020). ζ Potential as a Measure of Asphalt Emulsion Stability. Energy Fuels.

[44] Delahaije R.J.B.M., Wierenga P.A., van Nieuwenhuijzen N.H., Giuseppin M.L.F., Gruppen H. (2013). Protein Concentration and Protein-Exposed Hydrophobicity as Dominant Parameters Determining the Flocculation of Protein-Stabilized Oil-in-Water Emulsions. Langmuir.

[45] Liang A., Tang C.H. (2013). pH-dependent emulsifying properties of pea [*Pisum sativum* (L.)] proteins. Food Hydrocoll..

[46] Tangsuphoom N., Coupland J.N. (2005). Effect of heating and homogenization on the stability of coconut milk emulsions. J. Food Sci..

[47] Welch R.W., Wynne Griffiths D. (1984). Variation in the oil content and fatty acid composition of field beans (*Vicia faba*) and peas (*Pisum* spp.). J. Sci. Food Agric..

[48] Yoshida H., Saiki M., Yoshida N., Tomiyama Y., Mizushina Y. (2009). Fatty acid distribution in triacylglycerols and phospholipids of broad beans (*Vicia faba*). Food Chem..

